# Is exercise a therapeutic tool for improvement of cardiovascular risk factors in adolescents with type 1 diabetes mellitus? A randomised controlled trial

**DOI:** 10.1186/1758-5996-2-47

**Published:** 2010-07-11

**Authors:** Mona A Salem, Mohammed A AboElAsrar, Nancy S Elbarbary, Rana A ElHilaly, Yara M Refaat

**Affiliations:** 1 Department of Pediatrics, Faculty of Medicine, Ain Shams University, Cairo, Egypt; 2 Department of Physical Medicine, Rheumatology & Rehabilitation, Faculty of Medicine, Ain Shams University, Cairo, Egypt

## Abstract

**Background:**

Type 1 diabetes mellitus (T1DM) is associated with a high risk for early atherosclerotic complications especially risk of coronary heart disease.

**Objective:**

To evaluate the impact of six months exercise prgram on glycemic control, plasma lipids values, blood pressure, severity and frequency of hypoglycemia, anthropometric measurements and insulin dose in a sample of adolescents with T1DM.

**Research design and methods:**

A total of 196 type 1 diabetic patients participated in the study. They were classified into three groups: Group (A) did not join the exercise program(n = 48), group (B) attended the exercise sessions once/week (n = 75), group (C) attended the exercise sessions three times/week (n = 73). Studied parameters were evaluated before and six months after exercise programe.

**Results:**

Exercise improved glycemic control by reducing HbA1c values in exercise groups (P = 0.03, P = 0.01 respectively) and no change in those who were not physically active (P = 0.2). Higher levels of HbA1c were associated with higher levels of cholesterol, LDL-c, and triglycerides (P = 0.000 each). In both groups, B and C, frequent exercise improved dyslipidemia and reduced insulin requirements significantly (P = 0.00 both), as well as a reduction in BMI (P = 0.05, P = 0.00 respectively) and waist circumference(P = 0.02, P = 0.00 respectively). The frequency of hypoglycemic attacks were not statistically different between the control group and both intervention groups (4.7 ± 3.56 and 4.82 ± 4.23, P = 0.888 respectively). Reduction of blood pressure was statistically insignificant apart from the diastolic blood presure in group C (P = 0.04).

**Conclusion:**

Exercise is an indispensable component in the medical treatment of patients with T1DM as it improves glycemic control and decreases cardiovascular risk factors among them.

## Background

Type 1 diabetes mellitus (T1DM) is associated with a high risk for early atherosclerotic complications. Risk of coronary heart disease is fourfold (in men) to eightfold (in women)more excessive in patients with TIDM as compared with that for the general population [1]. Microvascular disease affects the tissues resulting in ischaemia. This microvascular disease is both structural (thickened basement membrane, capillary wall fragility and thrombosis) and functional (vasomotor neuropathy with defective microcirculation and abnormal endothelial function. Ischaemia also results from macrovascular disease (atherosclerosis) brought about by the direct metabolic injury, changes in vascular reactivity and accumulating endothelial injury [2]. Development of atherosclerotic lesions in healthy subjects begins upon childhood. In children with type 1 diabetes who had died an unnatural death, an asymptomatic increase in the intima-media thickness of the common carotid artery was found [3].

It has been demonstrated that high levels of serum total cholesterol, triglycerides, LDL, VLDL, glycosylated hemoglobin (HbA1c), microalbuminuria, hypertension, low concentration of HDL and increased body mass index (BMI) are significantly associated with coronary heart disease [4].

This study was designed primarily to evaluate the impact of six months exercise program on glycemic control, plasma lipids values, blood pressure, frequency of hypoglycemia, anthropometric measurements and insulin requirements in a sample of adolescents with type 1 diabetes mellitus.

## Patients and methods

This prospective randomized control study was conducted at Diabetes Specialized Clinic Children's Hospital, Ain Shams University from February 2009 to November 2009. A total of 196 patients (75 males, 121 females) were recruited for this study. The patients were regular attendees at the Clinic which cares for more than 1500 children, adolescents, and young adults with T1DM. To be eligible for the study patients had to satisfy the following criteria: 12-18 years old, diabetic with T1DM for at least 3 years and glycosylated hemoglobin (HbA1c) ≥7.5 (%)for the last six months before participating in the study.

Exclusion criteria included: significant diabetic complications limiting exercise like (diabetic foot, retinopathy, severe neuropathy), uncontrolled hypertension, diabetic keto-acidosis (DKA), severe hypoglycemia within the past 3 months, patients on lipid lowering therapy.

Patients' mean age was 14.78 ± 2.31 years, their disease duration ranged between 3 years and 10 years with a mean of 4.6 ± 1.9 years. Patients included used human insulin in a dose ranging from 0.65 to 2.1 IU/Kg/day with a mean of 1.19 ± 0.36 IU/Kg/day. All participants were on intensive insulin therapy.

Eligible patients were simply randomly assigned into two exercise intervention groups or in one control group as follows: Group (A) the control group, consisted of 48 patients that did not join the exercise program, group (B) consisted of 75 patients that attended the exercise sessions once/week and group (C) consisted of 73 patients that attended the exercise sessions three times/week. The attending diabetologist who randomly grouped the patients were different from those conducting the study. Of the 194 participants who were evaluated and joined the exercise program, only 148 patients completed the intervention arm of the study. Subjects participated after written information and informed consent from their parents. The study was approved by the Ethical Committee of Ain Shams University before commencement.

## Methods

All subjects underwent the following:

### Detailed Questionnaire that included

Complete history taking including their ages, duration of diabetes, complications, insulin regimen, history of acute diabetic complications (hypoglycemic attacks, hyperglycemia and DKA, history of exercise(type, frequency, duration).

### Clinical assessment that included

Physical examination included: anthropometric measures; weight in kilograms (kg), height in centimetres (cm) were plotted against percentiles for age and sex according to Growth Charts. The body mass index (BMI) was first calculated as weight in kgs. divided by square of height in meters, then body mass index standard deviation score (BMI SDS) was calculated [5]. Waist circumference (WC) in cm was measured at the part of the trunk located midway between the lower costal margin and the iliac crest while the patient was standing. Additionally, blood presure (systolic & diastolic) in millimeters mercury (mmHg) was measured and recorded using an automatic sphygmomanometer in three different occassions after they were seated and allowed to rest for 15 minutes.

### Biochemical investigations

All patients were asked not to exercise for three days before sample taking and lipid profile was taken after an over night fasting (12 hours). About 5 ml of venous blood was withdrawn from a prominent superficial vein using a clean venepuncture with minimal stasis, taken in a tube with EDTA, centrifuged and frozen. High density lipoproteins (HDL-c) were measured by precipitation method. Triglycerides (TG) were measured by enzymatic-calorimetric method. Cholesterol was measured by enzymatic-end point method. Low density lipoproteins (LDL-c) were measured by calculation methods i.e. Friedwaled equation [6] as follows:

Dyslipidemia was defined as cholesterol >200 mg/dl, HDL-c <35 mg/dl, LDL-c >160 mg/dl, or triglycerides >150 mg/dl [7]. The remaining part of sample was used to measure glycosylated hemoglobin (HbA1c) using the quantitative calorimetric determination of glyco- haemoglobin in whole blood, Stambio glycohaemoglobin procedure number 0350. Patients were asked to measure mean random blood glucose (MRBG) using Accu-Chek meter and reactive strips containing glucose dyeoxidase-reductase (Roch Diagnostics, Inc., Indianapolos) before and 6, 12 and18 hrs after they had excersised and to keep a detailed record in a diary for their previously mentioned blood glucose measurements. Baseline assessment of lipid profile, HbA1c measures and clinical parameters were performed before patients were randomized and occurred 1 to 14 days before classes started and were re-evaluated 6 months later after the exercise program.

### The exercise program

#### Groups B and C attended this program for 6 months that consisted of

A)An exercise training program according to Hornsby & Albright [8] under supervision of our physiatrist who directed the exercise intervention arm of the study. It consisted of: 1) Aerobic exercises either cycling/treadmill. The session consisted of five minutes warming up period followed by twenty minutes of training period followed by five minutes of cooling down period. During exercise the heart rate was monitored to ensure maximal safe exercise limit. To adjust the workload target heart rate range(THRR) was used 'THRR = 220-age in years ×(65-85%). If the heart rate of the patient exceeded the upper limit of THRR, then exercise intensity was slowed down but if it was less than the lower limit of THRR then exercise intensity was increased. The intensity of the exercise period was gradually increased according to THRR as the patients became conditioned during training.

2) Anaerobic exercises was performed on the treadmill which was achieved by interval training with intense short duration activity of 1-2 minutes with THRR reaching 85-95% of maximal heart rate. 3) Leg extension & leg curl exercises were done using weight machines, the goal was to increase number of repetitions and improve performance. Progressive resistive exercises (PRE) (DeLorme technique)was used. The 10 repetition maximum (RM) was first determined for each patient. 10RM was assessed for bilateral leg extension and leg press using weight machines. The weight machines were adjusted to meet the requirements of each subject. The 10 RM refers to the greatest amount of weight that could be lifted or pushed 10 times, the patient then performed one set of 10 repetitions at 50% of the 10 RM, a second set of 10 repetitions at 75% of the 10 RM, and final set of 10 repetitions at the full 10 RM. All three sets were performed at each session for about 10 minutes with a brief rest period (of 2 minutes) in between sets. Every week, a new 10 RM was determined as strength increased. 4)Different free strength and endurance exercises(done for 10 minutes), were performed as well in the session to help strengthen the foot and leg muscles. Exercises included bent calf knee raises, standing calf lifts and toe curls. Exercises were repeated 10 times (one set) and number of sets increased gradually through the program by one set every other session.

5) Flexibility exercises (for 5 minutes) were included which was achieved by stretching, the goal was to maintain and increase range of motion (ROM) and improve gait.

6) Neuromuscular exercises was done (for 5 minutes) by co-ordination exercises which were used to improve balance & co-ordination. The patients performed repetitions of heel to chin movements (10 repetitions) and heel to toe walking as well as walking sideways with crossing over feet, the patients did the different walks for five minutes each.

B) Balance exercise regimen which was performed according to Richardson et al. [9] on a firm surface for 10 minutes. They included warm up (ankle ROM with writing alphabet in the air with each foot), neck flexion, rotation and stretching while seated with eyes open then closed, foot inversion and eversion and bipedal wall slides with maximum knee flexion at 45Ëš. They started with one set of 10 repetitions and increased gradually to 3 sets after 6 sessions. Strengthening to upper body using abdominal crunch exercises and back lifts with extended arms and using overweight pulley were also preformed. Patients were given a booklet illustrating the exercises needed to help with their independent practice. In the control group, patients were instructed not to undertake any formal exercise or change their physical activity level during the study period.

### Statistical analysis

All statistical analysis were performed using the SPSS 10th version of Windows (Statistical Package for the Social Sciences). Descriptive statistics: mean, standard deviation, minimum, maximum and range of numerical data. Frequency and percentage of non-numerical data, paired sample Student's t test was used to test the difference between pre and post of some parameters (for continuous variables). Independent sample Student's t test was used to test the difference between two groups (for continuous variables). Chi square test was used to compare between groups regarding non numerical variables. Correlation (Pearson correlation coefficient r) assessing strength and direction of the linear relationship between two variables. One way ANOVA test (F) was used to test difference between more than two means. The probability was considered to be statistically significant at P value < 0.05.

## Results

One hundred and forty eight patients (76.3%) adhered to the supervised exercise sessions. The clinical characteristics of the enrolled patients before joining exercise program are demonstrated in table 1. The statistical analysis revealed that all of the pretreatment parameters were well balanced between the three groups of patients. The mean duration of DM was 4.6 ± 1.9 years, mean HbA1c level was 8.7%, mean age of patients was almost comparable (P = 0.94). As shown in table 1, there were no significant differences at the base line between the groups regarding their frequency of hypoglycemia, anthropometric measurements, blood pressure, insulin doses and HbA1c levels. In group C, the mean duration of DM was longer, in addition LDL-C, TG and cholesterol were higher than in groups A and B.

**Table 1:** Descriptive data of patients enrolled in the study before joining exercise program.

Variable	Group A	Group B	Group C	P-value
no.	48	75	73	
Age (years)	15 ± 2.35	14.7 ± 2.2	14.5 ± 2.4	0.94
D.M. duration (years)	4.9 ± 1.9	3.6 ± 1.8	5.5 ± 2	0.04
Hypoglycemia (times/month)	4.5 ± 3.43	4.8 ± 5.3	4 ± 4.75	0.88
weight (percentile)	64.2 ± 25.7	77.6 ± 20.9	77.4 ± 16.7	0.95
BMI (SDS)	0.7 ± 0.5	0.77 ± 0.34	0.69 ± 0.48	0.99
SBP (mmHg)	35 ± 17.4	67.75 ± 19.9	68.2 ±18.7	0.09
DBP (mmHg)	49 ± 21.3	61.5 ± 21.1	57.6 ± 24.4	0.79
Waist circumference (cm)	64.5 ± 11.2	64.6 ± 9.1	69.7 ± 12.9	0.37
Insulin dose (unit/Kg/day)	1.2 ± 0.37	1.16 ± 0.3	1.2 ± 0.4	0.93
HbA1c (%)	8.3 ± 2.1	8.9 ± 1.4	8.9 ± 1.6	0.55
LDL-C (mg/dl)	94.3 ± 22.8	101.2 ± 37.8	132.2 ± 34	0.01
HDL-C (mg/dl)	39.8 ± 5.4	46.3 ± 12.8	36 ± 5.5	0.01
TG (mg/dl)	151.9 ± 31.8	121.7 ± 41.2	176 ± 37.1	0.001
Cholesterol (mg/dl)	195 ± 21.5	171.8 ± 55.1	197 ± 27.7	0.18

### Anthropometric parameters

#### Weight (Percentile)

At the end of six months intervention protocol; the participants in group B and C had average weight percentiles that fell but still within the normal recommendations after their exercise sessions (P = 0.001, P = 0.000 respectively). A significant increase in weight percentile was noted in the control group (p = 0.03) table 2.

**Table 2:** Weight (percentile) in the studied groups before and after 6 months of exercise protocol.

	*Before*		*After*			
	Range	Mean ± S.D	Range	Mean ± S.D	t-value	P-value
Group (A)	25-97	64.2 ± 25.7	25-97	74.9 ± 22.5	-2.25	0.03
Group (B)	25-97	77.6 ± 20.9	25-90	58 ± 25.1	7.34	0.001
Group (C)	50-97	77.9 ± 16.7	25-90	47 ± 23	7.27	0.000

#### Body mass index (SDS)

The group assigned to perform more frequent exercises(group C) showed a significant reduction in BMI (BMI(SDS) decreased from 0.69 ± 0.48 to 0.5 ± 0.38, P = 0.001) more than the group assigned to perform once/week(group B) exercise (BMI(SDS) decreased from 0.77 ± 0.34 to 0.68 ± 0.3, P = 0.05). In group A no significant change in BMI was observed (0.7 ± 0.5 to 0.78 ± 0.5, P = 0.78).

### Waist circumference

The data also show that after training there were statistically significant reduction in the waist circumference (W.C.) in both groups B & C as it decreased from (64.6 ± 9.1 to 59.9 ± 8.2 cm, P = 0.02) and from (69.7 ± 12.9 to 61.5 ± 10.4 cm, P = 0.00 respectively), but with no difference demonstrated in group A (64.5 ± 11.2 to 64.3 ± 11.3 cm, P = 0.66).

### Blood pressure in percentile

There were no change in systolic & diastolic blood pressure in all groups during the course of six months intervention protocol (table 3). However, the physically active group C showed significant reduction in pre-exercise diastolic blood pressure between the beginning and end of the exercise protocol(P = 0.04)

**Table 3:** Blood pressure (systolic & diastolic percentile) in the studied groups before and after 6 months of exercise

	*Before*		*After*			
	Range	Mean ± S.D	Range	Mean ± S.D	t-value	P-value
Systolic Group (A) diastolic	25-75	35 ± 17.4	25-90	44 ± 24	-1.9	0.29
	25-90	49 ± 21.3	25-90	50.4 ± 21.3	-0.59	0.9
Systolic Group (B) diastolic	50-97	67.75 ± 19.9	50-90	66.7 ± 16.7	0.36	0.52
	25-95	61.5 ± 21.1	25-90	57.6 ± 16.5	1.44	0.07
Systolic Group (C) diastolic	50-97	68.2 ± 18.7	50-90	62 ± 14.4	1.07	0.3
	25-95	57.6 ± 24.4	25-95	50.8 ± 22.3	9.14	0.04*

### Insulin dosage

Of particular significance was the result of reduction in insulin dose after performing exercise protocol; as the mean insulin requirements decreased significantly in both groups B & C from (1.16 ± 0.3 to 1.1 ± 0.26 units per Kg per day, P = 0.002)and from (1.2 ± 0.4 to 0.9 ± 0.3 units per Kg per day, P = 0.00 respectively) and no change in patients who did not adhere to exercise protocol (1.2 ± 0.3 to 1.4 ± 0.42 units per Kg per day, P = 0.49).

### Hypoglycemia

The frequency of hypoglycemic attacks were comparable at the beginning of the study between the control arm and the intervention arm (4.5 ± 3.43 and 4.4 ± 4.92, p = 0.765 respectively). After six months of the exercise program; the frequency of hypoglycemic attacks were not statistically different between both study arms (4.7 ± 3.56 and 4.82 ± 4.23, P = 0.888 respectively).

### HbA1c level

**Figure 1:**
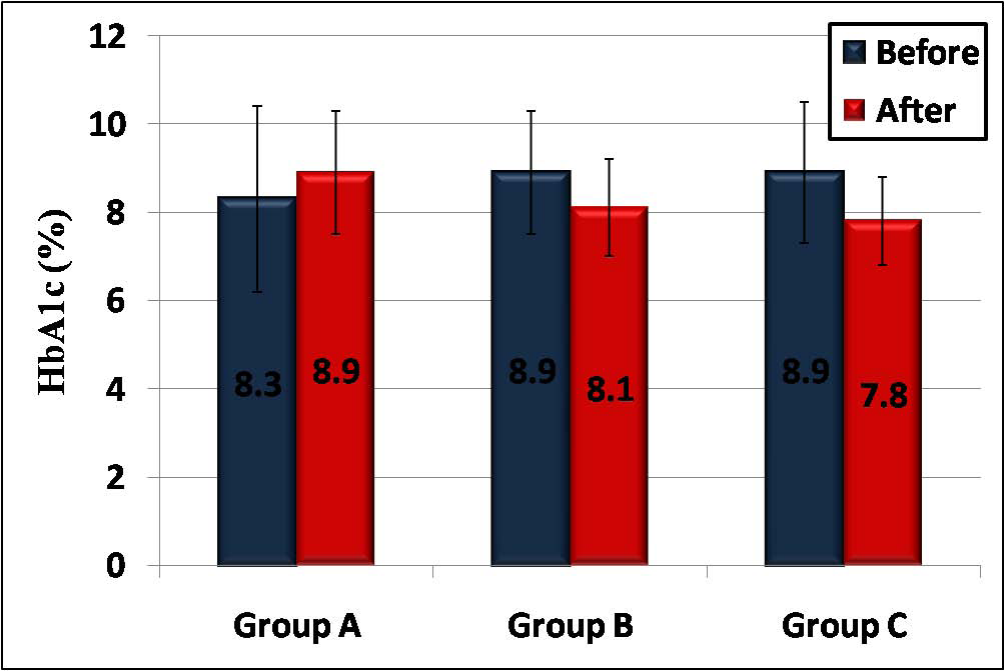
**A reduction in mean level of HbA1c in both groups B & C (p = 0.03, 0.01 respectively), while no change in group A(p = 0.2)**.

There were significant improvements in the mean HbA1c readings (fig.1) post exercise in both groups B and C (P = 0.03, P = 0.01 respectively). However, the greater reduction was noted in patients who performed regular exercise 3 times/week, and at the end of the study their HbA1c levels were significantly lower than the group that performed once/week exercise sessions (HbA1c decreased from 8.9 ± 1.6 to 7.8 ± 1% in group C and from 8.9 ± 1.4 to 8.1 ± 1.1% in group B). While mean HbA1c reading was not statistically different in those who had sedentary behavior (HbA1c 8.3 ± 2.1 at initiation of the study and 8.9 ± 1.4% at the end, P = 0.2), this effect was found in both sexes.

Higher levels of HbA1c were correlated with higher levels of cholesterol, LDL-c, and triglycerides before and after the exercise program (P = 0.000 each). While there were no significant correlation between HbA1c, DM duration(P = 0.97 for both) and HDL-c neither before nor after the exercise program (P = 0.54, P = 0.66 respectively) table 4.

**Table 4:** Correlation between HbA1c and each of these measurements in the studied groups before and after 6 months of the exercise program.

	*Before*		*After*	
Variables	r	P	R	P
Triglecrides	0.545	0.00**	0.531	0.00**
Cholesterol	0.513	0.00**	0.598	0.00**
LDL-c	0.446	0.00**	0.33	0.03*
HDL-c	-0.1	0.54	-0.071	0.66
DM duration	-0.006	0.97	-0.005	0.97

r = correlation coefficient

There were significant positive correlation between HbA1c and TG, cholesterol and LDL-c before and after the exercise program. While there were no significant correlation between HbA1c and DM duration, HDL-c neither before nor after the exercise program.

### Lipid profile

**Figure 2:**
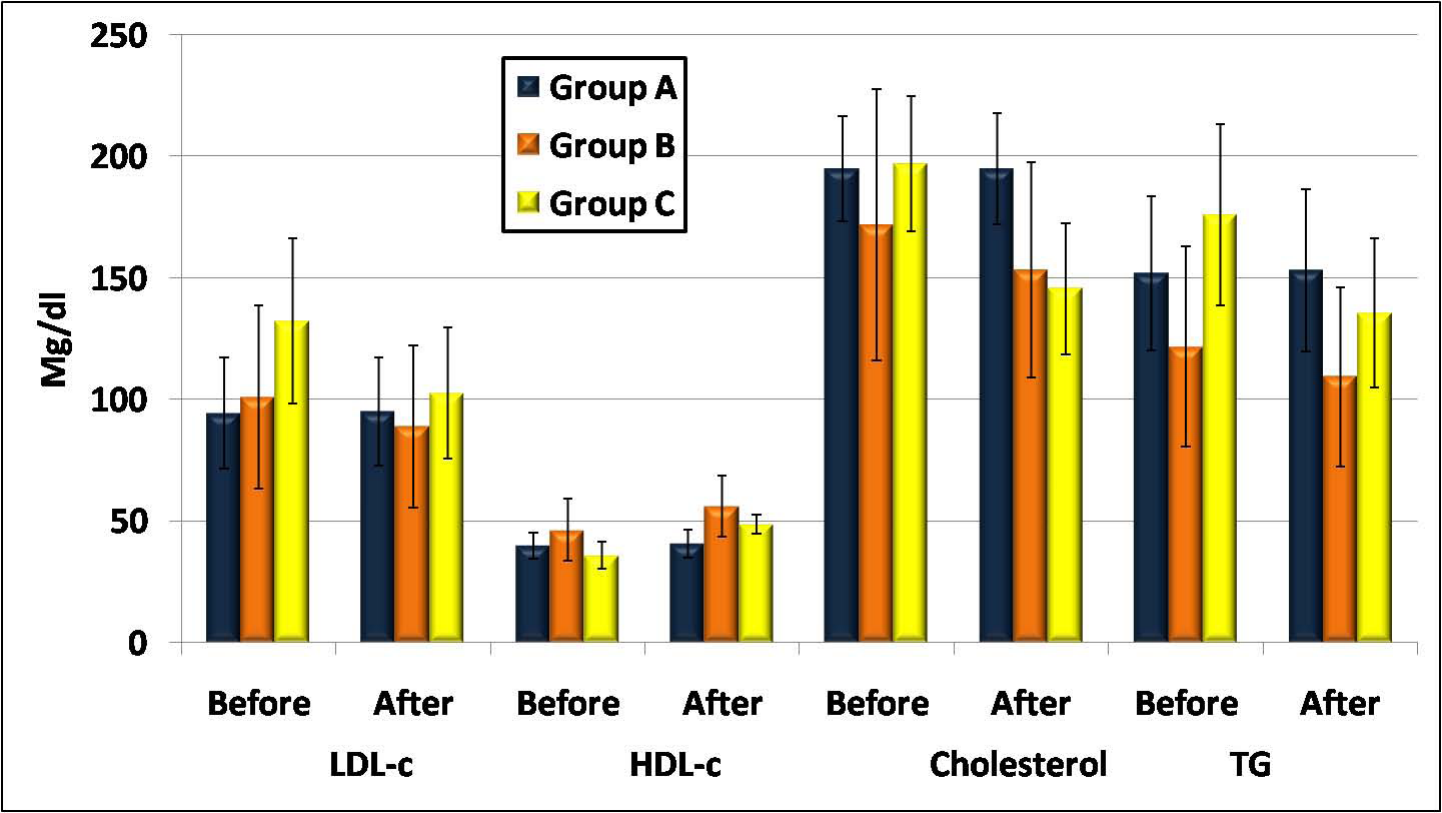
**Frequent exercise was associated with statistically significant decrease in the lipid profile levels of LDL-c, TG, & cholesterol, and statistically significant increase in HDL-c in both groups B&C**.

Frequent exercise was associated with statistically significant decrease in the lipid profile levels of LDL-c, TG, & cholesterol, and statistically significant increase in HDL-c in both groups B&C(fig.2).

Reduction in LDL-c was noticed in both groups B & C; as it decreased from 101 ± 37.8 to 89 ± 33.4 mg/dl and from 132.2 ± 34 to 102.7 ± 27 mg/dl, P = 0.01, P = 0.001 respectively, while no change in group A (94.3 ± 22.8 to 95 ± 22.37 mg/dl, P = 0.45).

Statistically significant increase in HDL-c in both groups B & C was observed; as it increased from 46.3 ± 12.8 to 56.1 ± 12.5 mg/dl and from 36 ± 5.5 to 48.6 ± 3.9 mg/dl P = 0.01, P = 0.00 respectively, while no change in HDL-c level was observed in group A (39.8 ± 5.4 to 40.8 ± 5.8 mg/dl, P = 0.22).

A reduction in total cholesterol in both groups B & C was statistically significant; as it decreased from 171.8 ± 55.7 to 153.4 ± 44.3 mg/dl and from 197 ± 27.7 to 145.6 ± 27 mg/dl, P = 0.01, P = 0.00 respectively, while no change in total cholesterol level was observed in group A (195 ± 21.5 to 194.9 ± 23 mg/dl P = 0.94).

The same was noticed in TG levels in both groups B & C as it decreased from 121.7 ± 41.2 to 109.4 ± 36.9 mg/dl and from 176 ± 37.1 to 135.6 ± 30.7 mg/dl P = 0.00 for both respectively, while no change in group A (151.9 ± 31.8 to 153.3 ± 33.4 mg/dl P = 0.49).

No significant correlation was found between DM duration and lipid profile levels (HDL-c, LDL-c, cholesterol, TG) (*r *= -0.08, P = 0.59, *r *= 0.026, P = 0.87, *r *= -0.018, P = 0.9, *r *= -0.008, P = 0.96 respectively).

Before attending the exercise program, dyslipidemia was seen in 52.5% of the patients. Elevations of triglycerides and of cholesterol were the most frequent types of dyslipidemia (50%, 45% respectively) followed by decreased HDL-c(22.5%)and elevated LDL-c(15%).

After 6 months of exercise program the percentage of patients with dyslipidemia decreased to 37.5%, triglycerides, cholesterol, LDL-c & HDL-c decreased to (35%, 17.5%, 2.5% & 2.5% respectively). Multiple regression analysis showed that lipid profile was the most affected variable by exercise, cholesterol reduction followed by HDL-c elevation were the most affected types of lipids.

## Discussion

The effect of 6-month supervised exercise on cardiovascular risk factors was studied in a group of 196 adolescentts with type 1 diabetes mellitus. Despite the well-known benefit of physical activity in improving known risk factors for atherosclerosis, unfortunately this modality was ignored by parents and patients who fear the consequence of hypoglycemia, which can occur during, immediately after, or many hours after physical activity, making it one of limiting factors for its practice in children and adolescents with type 1 DM.

The frequency of hypoglycemia experienced by the intervention group was the same in comparison to the control group. Our results might be influenced by the fact that the exercise in this study is a planned one and patients do not exert any unscheduled activity. This will give the chance for an educated patient the ability to learn about his individual glucose response to exercise and eventually establish an individual strategy to prevent exercise-induced hypoglycemia. Framework to guide management of achieving blood glucose goals without excessive hypoglycemia during and after exercise can be accomplished through individualized meal planning, flexible insulin regimens and algorithms, self monitoring blood glucose and education promoting decision-making based on documentation and review of previous results.

Some studies [10-12] found that the frequency of exercise had no significant influence on the frequency of severe hypoglycemia or hypoglycemia with occurrence of seizure or loss of consciousness.

In their study in 2006, ***DirecNet group ***[13] found that in youth with T1DM, prolonged moderate aerobic exercise results in a consistent reduction in plasma glucose and the frequent occurrence of hypoglycemia when pre-exercise glucose concentrations are <120 mg/dl. Moreover, treatment with 15 g of oral glucose is often insufficient to reliably treat hypoglycemia during exercise in these youngsters.

Of particular significance is the result of reduction in insulin requirements after performing exercise. The authors speculated that patients tend to reduce their insulin dosage to prevent exercise induced hypoglycemia. Moreover, exercise acutely lowers the blood glucose concentration to an extent that depends on its intensity, duration and the concurrent level of insulinaemia [14]. It also increases insulin-stimulated glucose uptake in muscle, putting in mind that this effect is higher in trained muscle than in untrained muscle, leading to a reduction in the insulin requirement [15].

Our results are in agreement with that of ***Herbest et al. ***[12] who confirmed that RPA is associated with a lower insulin dosage as a long-term effect. Many other studies [16-18] found the same significant effect of regular physical exercise on the daily insulin dose.

Our study shows that HbA1c level is influenced by frequency of exercise, as it was lower in group C with more frequent exercise, this may be because physical activity is known to acutely reduce the blood glucose level and to increase insulin sensitivity [19]. However, ***Allen et al***. [20] have shown that the better control of glycemia (lower HbA_1c _level) due to regular exercise is related to more frequent mild and severe hypoglycemia and hence decreasing HbA1c levels. The reduction in HbA1c level over time is clinically significant, as the Diabetes Control and Complications Trial (DCCT) reported a 21% to 49% decreased risk for microvascular complications with every 1% decrease in HbA1c [21].

The relationship between physical fitness and HbA1c levels has been previously studied; however, the results were controversial. ***Herbst et al***. [22] studied the cardiovascular risk factors among 23, 251 patients (3-18 years) with type 1 diabetes, found that mean HbA1c was 7.9%. The frequency of RPA defined as physical activity performed regularly at least once a week for at least 30 minutes continuously for at least 1 year, excluding school sports ranged between 0 and 9 times/week (average 1.29 times/week). Of the patients, 44.7% were not physically active, 37.0% performed RPA 1-2 times/week, and 18.3% performed RPA 3 times/week. HbA1c was lower in patients with a higher frequency of RPA (*P *< 0.00001), Higher levels of HbA1c were associated with higher levels of cholesterol, LDL cholesterol, and triglycerides (*P *< 0.0001 each) and a lower level of HDL cholesterol (*P *< 0.01).

In addition, ***Herbst et al***. [12] who studied the relationship between the frequency of RPA and glycemic control in patients with T1DM aged 3 to 20 years revealed that HbA1c level was higher in the groups with less frequent RPA (8.4% in group RPA0 vs 8.1% in group RPA2; *P *< .001). This effect was found in both sexes and in all age groups (*P *< .001). Multiple regression analysis revealed that RPA was one of the most important factors influencing the glycosylated hemoglobin level. Other studies [23-25] also revealed that the more frequent exercise performed the lower HbA1c level.

Others [16] aimed to assess the effect of 6-month exercise program in obese children and showed that HbA1c level was decreased after the exercise program but not a statistically significant decrease, this may be due to the limited time of the program, or due to compliance of patients.

It is worth noting the study of ***Harmer et al***. [26] which aimed to investigate the effects of high intensity exercise (HIE) training on glycemia and acid-base regulation in type 1 diabetes, found that exercise training did not alter HbA1c in type 1 diabetic subjects (pre-exercise 8.6 ± 0.8%, post-exercise 8.1 ± 0.6%; *P *= 0.09). Furthermore, ***Ramalho et al***. [17] evaluated the effect of aerobic versus resistance training on metabolic control in type-1 diabetes patients and found that neither resistance nor aerobic training had improved glycated hemoglobin in type-1 diabetes patients. Some studies have failed to show the effect of exercise on HbA1c [27-29]. The controversial results might be because of the different methodological approaches or because of different number of studied groups.

In the current study, patients in the exercise group have gained more benefits from joining the exercise program. This group showed significant improvements related to lipid profile and found that frequent exercise was associated with statistically significant decrease in the lipid profile levels of LDL-c, TG, & cholesterol, and statistically significant increase in HDL-c. Exercise is considered as one approach for maintaining optimal lipid levels in addition it is a low-cost, non- pharmacologic intervention that is available to the vast majority of children and adolescents.

Our findings were consistent with the previous report by ***Herbst et al***. [22] who found that increasing frequency of RPA was associated with lower total cholesterol, LDL cholesterol, and triglycerides and higher HDL cholesterol. Moreover, ***George et al***. [30], ***Lehmann et al***. [31], ***Meyer et al***. [16], ***Valerio et al***. [32] proved the effect of exercise on plasma lipids and found that the frequency of exercise was associated with a significant decrease in the levels of LDL-c, cholesterol & TG, and increase in HDL-c level.

Where as ***Gordan et al***. [33] whom studied the effect of exercise therapy on lipid profile and oxidative stress indicators in patients with type 1 diabetes, found that 6-month exercise program significantly decreased total cholesterol (P value < 0.001) but didn't affect TG, LDL-c or HDL-c.

In their study in 2009, ***Leite et al***. [34] analyzed the effects of physical exercise and nutritional guidance on body composition, physical fitness, lipid profile and insulin resistance among obese adolescents and found after 12 weeks exercise program 3 times per week that TG decreased significantly (P < 0.001) and HDL increased significantly (P < 0.001) but total cholesterol & LDL had shown no significant decrease. In addition ***Rigla et al***. [18] who evaluated the effect of physical exercise (3-month exercise program) on blood pressure, the lipid profile, lipoprotein(a) and LDL-c in type 1 & 2 diabetic patients, presented an increase of HDL-c in type 1 patients (*P *< 0.05), while a decrease of LDL-C in type 2 patients (*P *< 0.01).

On the contrary, other studies [18,24,35] have failed to show the effect of exercise on lipid profile, this may be due to the limited time of the program, or due to compliance of patients. Also total cholesterol, LDL-cholesterol, TG and plasminogen activator inhibitor-1 (PAI-1) levels were lower in highly trained athletes group in relation to sedentary subjects (p < 0.01). These results indicate that lifestyle associated with high intensity and high volume exercise induces favorable changes in the lipid profile and PAI-1 levels, and this may reduce the risk of cardiovascular diseases [36].

Although weight and BMI of participants in this study before joining exercise program were normalized, yet they were towards the high normal side. This is important because, during puberty, adolescents have a higher risk for gaining weight. Moreover, there is an epidemic towards increase prevalence of overweight and obesity among children and adolescents. Exercise might keep the BMI or body weight low due to its lipolytic effect. At the end of the study; the average body weight was statistically different between the two intervention groups and the control group keeping the patients who performed exercise within 50^th ^percentiles and not causing severe weight loss that may have affected the growth and development of those patients in their growing ages. Accumulating evidence [37] suggests that physical activity may enhance weight loss and, in particular, weight maintenance thereafter to achieve and maintain ideal body weight and body composition when used along with an appropriate calorie controlled meal plan and an increase in calcium and vitamin D supplementation [38].

***Leite et al***. [34] and ***Kelley et al***. [35] proved that aerobic exercise improved percent of body fat, aerobic capacity & decreased body weight, BMI, WC significantly.

Whereas, other studies [17,18] found no significant change in BMI after exercise, but found a significant change in waist circumference after the exercise program.

Little difference was obseved concerning blood pressure either systolic or diastolic in the three groups after exercise program, this may be due to the young age of the patients as most of them were normotensive.

Others [18,22] found that the regular physical exercise did not affect the systolic blood pressure, but it decreased the diastolic blood pressure significantly (P value < 0.001).

In contrast, other studies [16,24,34] found that the systolic blood pressure was affected significantly (P value = 0.048) but the diastolic blood pressure was not affected by the exercise program.

Many studies used different aspects of exercise regimen. Lehman et al. [31] included well-controlled subjects with IDDM (n = 20; HbA1c = 7.6%) and they were engaged in a regular exercise program over a period of 3 months involving endurance sports such as biking, long-distance running, or hiking. Ramalho et al. [17] evaluated the effect of aerobic versus resistance training on metabolic control in type-1 diabetes patients. Thirteen non-active patients, ranging in age from 13-30 yrs, were submitted to a 12-week aerobic exercise (Group A, n = 7) or resistance training (Group B, n = 6) period. Durak et al. [23] studied on eight male type 1 diabetic subjects (mean +/- SD age 31 +/- 3.5 yr) and their program consisted of heavy-resistance weight training 3 days/wk for 10 wk, concentrating on the strengthening of major muscle groups through progressive resistance. Guffee et al. [11] evaluated eight volunteers with type 1 diabetes (aged 18.6 ± 2.1 years) that joined either the control rest protocol (CON) or intermittent high-intensity exercise protocol (IHE). In patients with type 2 diabetes, a few studies have looked at the combined effect of aerobic and resistance exercises [39-41]. However, our training program aimed to combine the different elements of an exercise regimen (aerobic, anaerobic, strengthening, neuromuscular and balance) and to address various aspects in type1 diabetes patient to help improve their physical function and quality of life. We aimed to improve blood glucose control and insulin sensitivity, reduce body fat, reduce stress, increase cardiovascular benefits aswell as increase muscle strength and endurance. Flexibility exercises and stretching was used to help prevent injury and maintain and or increase range of motion of joints. The neuromuscular and balance training part of our program with special emphasis on the lower limbs was unique and aimed to improve the coordination, balance and physical function of patients.

In conclusion our results implicated that exercise might greatly benefit many patients with diabetes by improving their metabolic profile, dyslipidemia, aiding in their weight loss and maintaining their blood pressure; these effects may translate into an improved vascular disease risk profile in adolescents with diabetes. The challenge is to develop strategies that allow individuals with T1DM to participate in activities that are consistent with their lifestyle and culture in a safe and enjoyable manner, and discouraging sedentary activities, especially time spent in front of the TV or computer monitor. Exercise should be an important component of self -care activity in the management of patients with T1DM.

## Abbreviations

BMI: Body mass index; Cm: Centimeter; GLUT4: Glucose transporter protein; HbA1c: Glycosylated haemoglobin; HIE: High intensity exercise; Kg: Kilograms; NEFA: Non-esterified fatty acids; PRE: Progressive resistive exercises; RM: Repetition maximum; RPA: Regular physical activity; SDS: Standard deviation Score; T1DM: Type 1 diabetes mellitus; THRR: Target heart rate range; U/kg/d: Unit/kilogram/day; WC: Waist circumference

## Competing interests

The authors declare that they have no competing interests.

## Authors' contributions

MAS participated in the concept, design of the study, data analysis, manuscript editing and review. MAA participated in the design, data analysis, manuscript editing and review.

NSE carried out literature search, clinical studies, data acquisition, data analysis, manuscript preparation, editing and review. RAE participated in design of the study, assigned and supervised the training programe, manuscript editing and review. YMR performed the lipid profile studies, data acquisition, statistical analysis

All authors read and approved the final manuscript.

## Authors' information

MAS, Professor of Pediatrics, Ain Shams University, Ahmed Saed St.Abbasia, Cairo, 11535, Egypt and vice president of the Egyptian Society of Pediatric Endocrinology & Diabetes (ESPED). MAA, Assistant Professor of Pediatrics, Ain Shams University, Ahmed Saed St.Abbasia, Cairo, 11535, Egypt. NSE, Lecturer of Pediatrics, Ain Shams University, Ahmed Saed St.Abbasia, Cairo, 11535, Egypt. RAE, Lecturer of Physical medicine, Rheumatology and Rehabilitation, Ain Shams University, Ahmed Saed St.Abbasia, Cairo, 11535, Egypt. YMR, Assistant Lecturer of Pediatrics, Ain Shams University, Ahmed Saed St.Abbasia, Cairo, 11535, Egypt
